# Using Ecological Systems Theory to Enhance Community Health Literacy

**DOI:** 10.3928/24748307-20241126-01

**Published:** 2025-01

**Authors:** Carolyn Dickens, Yolanda Suarez-Balcazar, Paula Allen-Meares, Eryn Brazil

## Abstract

**Background:**

The COVID-19 pandemic exacerbated long-standing disparities that many people in the United States experience due to their race and ethnicity and socioeconomic status.

**Brief Description of Activity:**

An outcry from several relevant stakeholders ignited a federal response from the Centers for Disease Control and Prevention (CDC) and the Department of Health and Human Services (HHS), who, among other entities, decided to address low health literacy (HL) in underserved communities. Evidence suggests that HL impacts under-resourced communities' understanding of health issues and whether they adhere to health guidelines.

**Implementation:**

This article aims to provide an ecological analysis of HL best practices, highlighting their role in community health during public health crises.

**Results:**

Although a vast amount of literature applies Ecological Systems Theory (EST) to understanding and addressing a range of issues impacting the health of communities, scarce literature applies EST to understanding HL interventions.

**Lessons learned:**

We discuss implications for public health efforts, concluding that Bronfenbrenner's Ecological Systems Theory is effective for grounding the development and implementation of best practices for promoting HL interventions. [***HLRP: Health Literacy Research and Practice*. 2025;9(1):e29–e36.**]

## Abstract (plain language summary)

**Plain Language Summary: **The Ecological Systems Theory (EST) provides a framework for understanding health literacy at different levels of impact on an individual. This perspective is ideal for advancing health literacy in communities disproportionately affected by social determinants of health. We postulate that efforts to advance health literacy grounded in the EST framework are more likely to promote a health-literate society and contribute to reducing health disparities.

Multiple public health initiatives have promoted evidenced-based health literacy (HL) practices to improve community and individual health outcomes (Liu et al., 2020). During the coronavirus disease 2019 (COVID-19) pandemic, it became clear that HL continued to be a critical public health issue, particularly in racially and ethnically diverse communities (Collins, 2022). As a result of the pandemic, the Centers for Disease Control and Prevention (CDC) and the U.S. Department of Health and Human Services (HHS) requested proposals for developing and implementing comprehensive educational training for health care providers focusing on improving skills to address HL. They recommended that educational programs for under-resourced communities include health literacy and strategies to establish trust and enhance communication (Hange et al., 2022). To act upon this recommendation, the CDC and HHS accepted proposals targeting HL and cultural relevance/humility in addressing racial disparities (Collins, 2022).

Health literacy is a widely discussed concept and has experienced significant evolution since its introduction 30 years ago (Liu et al., 2020). The CDC distinguishes “personal health literacy” from “organizational health literacy.” The former is “the degree to which individuals have the ability to find, understand, and use information and services to inform health-related decisions and actions for themselves and others” (CDC, 2024b). Organizational health literacy is “the degree to which organizations equitably enable individuals to find, understand, and use information and services to inform health-related decisions and actions for themselves and others” (CDC, 2024b).

The CDC stated that health communication needs to involve greater cultural and community focus to engage residents in public health efforts (Airhihenbuwa et al., 2020; CDC, 2024a). If the cultural norms of a community don't align with the values of health care systems or public health initiatives, communities will have difficulty accessing the information or trusting the information provided (Airhihenbuwa et al., 2020). When health care providers take the initiative to involve patients in developing their care plans in a culturally sensitive manner, their patients' HL improves (CDC, 2024a).

The federal financial support for HL interventions during COVID-19, validated the need for health literacy interventions to be developed in both public health systems and health care organizations. COVID-19 highlighted that HL is pivotal for individuals to dismiss misinformation and understand public health safety measures during public health emergencies (Nutbeam & Lloyd, 2021). The authors of this article propose a systematic approach using a theoretical framework to assist in future initiatives addressing HL.

Grounded in the Ecological Systems Theory (EST), this article aims to analyze and make recommendations for HL interventions at different levels of the theoretical framework—microsystem, mesosystem, exosystem, macrosystem, and chronosystem (Bronfenbrenner, 1979). Due to the expanding nature of social media and technology on health equity, the authors include interventions addressing media literacy and e-literacy as well. The authors follow the analysis by discussing the implications for addressing HL.

## Brief Description: Covid-19 Amplified Health Disparities and Health Equity Issues

The COVID-19 pandemic, a global public health tragedy, exacerbated health disparities, impacting communities already experiencing economic and social hardships, such as Black, Hispanic, Latino/a/e, and Indigenous communities (Andraska et al., 2021). The Hispanic/Latino population suffered tremendously during the pandemic as they were more likely to take public transportation, live in high-density environments, and work in the service sectors (Andraska et al., 2021). Some Hispanic and Latino/a/e immigrant communities and people who are undocumented experience unique obstacles in safely accessing the health care system, due to issues surrounding their immigration status, language barriers, and cultural differences (Andraska et al., 2021). Black communities also faced a disproportionate impact during the pandemic as they were less likely to be insured, lacked access to testing, experienced racism, worked in essential jobs, and had underlying health conditions (Vasquez Reyes, 2022). COVID-19 similarly impacted rural populations due to limited access to COVID-19 testing and higher poverty rates (Mueller et al., 2021). People who live in rural areas typically have less access to health care, forcing residents to rely heavily on telehealth (Mueller et al., 2021).

Unfortunately, the role of misinformation in promoting vaccine hesitancy was evident during the pandemic (Zhao et al., 2023). Vaccine hesitancy was associated with lower education, economic status, and minority status (Zhao et al., 2023). To address the increasing influence of social media and the spread of misinformation, the concept of “media literacy,” an evolving concept, should be included in the concept of health literacy (Levin-Zamir et al., 2018). Media literacy is the ability to identify health-related content in various media types and critically analyze the content (Levin-Zamir, 2018). Social media and networking platforms are increasingly popular sources of health information, but often, the content has not been vetted and can become a source of health misinformation (Suarez-Lledo & Alvarez-Galvez, 2021). The pandemic highlighted the use of social media to disseminate health misinformation, resulting in the under-utilization of proven treatments such as masks and vaccines and promoting the use of unapproved and dangerous treatments for COVID-19 (Montagni et al., 2021).

E-literacy became more relevant during the pandemic, with 85% of the U.S. population reporting that they looked for medical information online (Sheon & Khoong, 2024). E-literacy is an individual's level of competence and understanding in using information technology and digitalized information (Levin-Zamir et al., 2018). Lack of access to the internet and reliance on smartphones is associated with minorities, lack of college education, and a household income of less than $50,000 (Sheon & Khoong, 2024). The pandemic revealed disparities in the use of telehealth among older adults, migrants, and people of color, demonstrating the additional support required to access their health care provider (Lane et al., 2023). Past studies have shown that older patients, patients with lower education, and those from racial and ethnic minority groups are less likely to register for a patient portal or use digital functions like online medication refills (Valdovinos et al., 2022). Using interpreters, another e-literacy skill, between health care providers and non-native-speaking patients is necessary for the best quality of care and culturally competent care (Heath et al., 2023). However, the availability of professional interpreters is limited, with health care providers sometimes using no interpreter or ad-hoc interpreters (Heath et al., 2023). Online interpreters are an option but typically are not used, and reasons cited by health care providers have been the time commitment and technical difficulties with interpretation technology (Ortega et al., 2020). E-literacy is a critical concept that needs to be addressed among health care workers and non-native-speaking communities when developing interventions to enhance health literacy.

## Implementation: An Ecological Systems Theory Analysis of Health Literacy and Recommended Interventions

EST examines the interaction between the individual and their cultural, sociopolitical, and economic environments, emphasizing the relationships formed by interacting levels of influence (Bronfenbrenner, 1979). Bronfenbrenner's EST has been utilized to understand and address health disparities and promote health. The World Health Organization and the CDC's models for social determinants of health are based on Bronfen-brenner's original theory (CDC, 2022). Health literacy is associated with social determinants of health, whereby populations with low HL are more likely to experience employment insecurity, limited lifetime income and education, and other factors (Nutbeam & Lloyd, 2020). Social determinants of health and EST both emphasize the importance and influence of environmental factors on an individual's HL.

**Figure 1.**
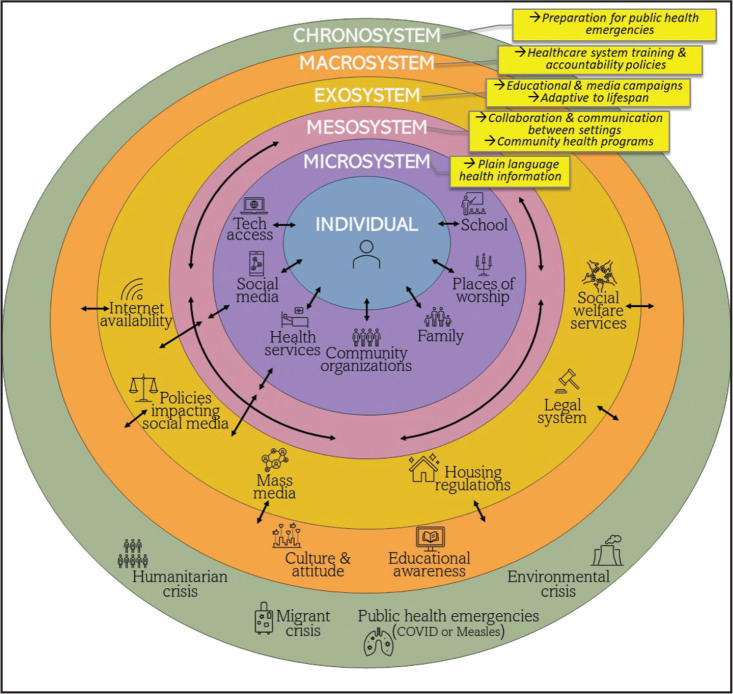
Ecological systems theory and examples.

Bronfenbrenner's EST comprises of five nested levels that influence an individual's behavior (**Figure 1**). The EST can be visualized by imagining an individual's HL at the center of five concentric circles: the microsystem, mesosystem, exosystem, macrosystem, and chronosystem. The EST stresses interdependency and interaction among the different levels. However, the EST recognizes the importance of the individual at the center of the nested levels.

The microsystem is the first level impacting the individual, defined as the immediate environment surrounding the individual (Bronfenbrenner, 1979; Jaeger, 2016). Specifically, this level focuses on direct interactions between individuals and their immediate environment, such as significant others, siblings, health care providers, and friends (Bronfenbrenner, 1979; Jaeger, 2016). The immediate environment can influence an individual's access to health care and their beliefs and values relating to health. Interventions targeted towards the microsystem should be directed at health information and materials that use simple language, contain visuals, and are available in the primary language spoken by clients and members of their immediate environment. HL interventions using plain language and easy-to-understand information should also target the family, places of worship, and school (Liu et al., 2020). Government agencies and health care organizations should implement HL interventions targeting social media platforms due to their immediate impact on the individual. The efforts targeted at this level should include educating families, schools, and places of worship about detecting misinformation and understanding where to find trusted health information on social media or the internet (Montagni et al., 2021). Health literacy efforts in communities must educate individuals and communities to detect misinformation (Montagni et al., 2021). Individuals should be assessed to determine whether they can properly access technology via Wi-Fi and computers versus cell phones. It is essential to determine whether the individual can understand the usability of digital tools such as electronic medical systems, Google Translate, telehealth, and health apps (Levin-Zamir et al., 2018). Digital navigators have shown promise in educating communities about internet access and using computing devices (Wang et al., 2024).

The next level is the mesosystem, which refers to interactions between various microsystems. Examples of the mesosystem include the interactions between family members and health care providers. Other examples included in the mesosystem are relationships between family members, peer relationships, and community connections. The mesosystem is particularly important because it exerts an immediate and direct influence on the individual, creating a coordinated support network (Mullen et al., 2018). HL interventions targeting this domain aim to improve collaboration and communication between various settings, such as schools, places of worship, health care facilities, and community organizations. Social media communities that incorporate groups of people would be included in the mesosystem. Interventions targeting the mesosystem can benefit from the interdependent relationships between different microsystems to facilitate positive change. Health literacy workshops would be appropriate for professionals in various health systems or public health organizations. Specifically, the meso-system level requires improved integration of technologies, institutions, and organizations to promote better health outcomes for a community.

The exosystem, the third level in EST, involves formal and informal social structures likely to influence the mesosystem directly or indirectly. The exosystem level is about the environment and the broader societal context (Bronfenbrenner, 1979; Crawford, 2020). This level is characterized by the external influences shaping an individual's life through policies and societal norms. The exosystem level highlights the impact of external settings and systems on an individual, such as education, the social media industry, technology, and social services. Due to the difficulty in monitoring or censoring health misinformation on social media and networking platforms, government or health care organizations should create social media campaigns to disseminate accurate information (Zhao et al., 2023). Educational programs offered through social media platforms (TikTok, YouTube, Facebook) and traditional media platforms (TV, radio, and newspapers) would make the public aware of the negative influence of chatbots, AI, and misinformation. Public educational programs targeting health misinformation might include “flagging” a trusted social information platform. Public policies at this level would include equitable internet access for specific populations, especially those in rural and poverty-segregated communities (Sheon & Khoong, 2024). The EST exosystem also recognizes the importance of the lifespan perspective: HL interventions should adapt to the evolving needs of individuals at different life stages.

The fourth level, the macrosystem level, comprises cultural norms, societal policies, and systems that impact the individual. For example, socioeconomic status exerts macro-level influence on individuals—with people with higher socioeconomic status having better health outcomes than those with lower socioeconomic status (Liu et al., 2020). Culture and language are significant at this level as they continually shape and influence individuals (Crawford, 2020). Cultural beliefs can directly impact a person's HL, as culture shapes an individual's beliefs, values, and attitudes toward health (Jaeger, 2016). While culture and language are influential at the microsystem level, they are also factors at the macrosystem level due to their multi-level and wide-reaching impact.

Language, religion, and culture influence access to quality health care in communities that don't speak English (Liu et al., 2020). Macrosystem-level interventions frequently overlap with exosystem interventions and may include policies requiring health care professionals to complete HL training and systems of care—like hospitals—becoming health-literate organizations with ongoing training and accountability. Exosystem interventions would also include the CDC/HHS funding supporting community health workers' training in health literacy, cultural humility, and digital literacy.

The fifth level, the chronosystem, relates to shifts and transitions over a lifetime—for example, historical events such as the pandemic fall within the chronosystem. COVID-19 was a historical event that broadly impacted society and communities. Other examples of public health events include measles outbreaks, the migrant and refugee crisis, and artificial intelligence (AI). Interventions at this level should be considered preparatory to prepare populations and public health organizations for future emergencies.

## Results

Health literacy interventions have evolved to meet the shifting landscape of health-seeking behavior, technology, and demographic factors. The changing technical and social media landscape during the pandemic highlighted the importance of including e-literacy and media literacy interventions throughout the EST to promote public health. This resulted from (1) the proliferation of e-health services and (2) the spread of health misinformation through social media platforms. Analysis of HL interventions throughout all levels of the EST provides opportunities to improve public health (**Table 1**). The strategies for interventions in the table are not all-inclusive and additional interventions are developed by scientists and policymakers in a consistent manner.

**Table 1:**
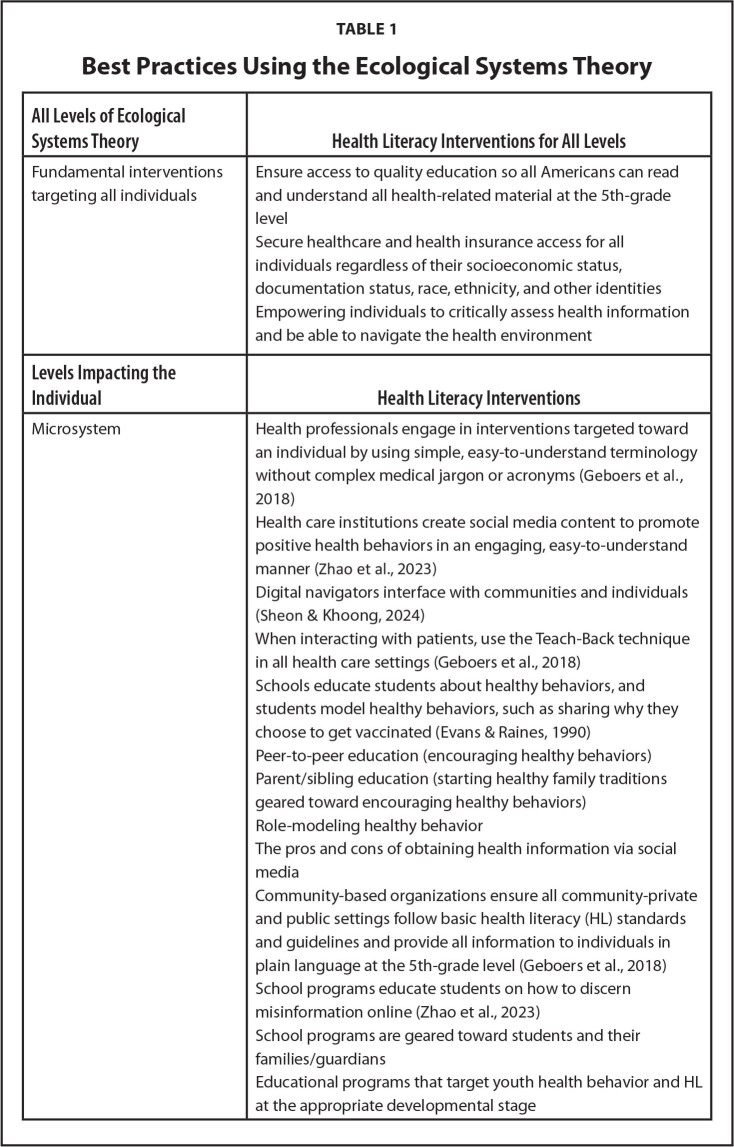
Best Practices Using the Ecological Systems Theory

**All Levels of Ecological Systems Theory**	**Health Literacy Interventions for All Levels**

Fundamental interventions targeting all individuals	Ensure access to quality education so all Americans can read and understand all health-related material at the 5th-grade level
	Secure healthcare and health insurance access for all individuals regardless of their socioeconomic status, documentation status, race, ethnicity, and other identities
	Empowering individuals to critically assess health information and be able to navigate the health environment

**Levels Impacting the Individual**	**Health Literacy Interventions**

Microsystem	Health professionals engage in interventions targeted toward an individual by using simple, easy-to-understand terminology without complex medical jargon or acronyms (Geboers et al., 2018)
	Health care institutions create social media content to promote positive health behaviors in an engaging, easy-to-understand manner (Zhao et al., 2023)
	Digital navigators interface with communities and individuals (Sheon & Khoong, 2024)
	When interacting with patients, use the Teach-Back technique in all health care settings (Geboers et al., 2018)
	Schools educate students about healthy behaviors, and students model healthy behaviors, such as sharing why they choose to get vaccinated (Evans & Raines, 1990)
	Peer-to-peer education (encouraging healthy behaviors)
	Parent/sibling education (starting healthy family traditions geared toward encouraging healthy behaviors)
	Role-modeling healthy behavior
	The pros and cons of obtaining health information via social media
	Community-based organizations ensure all community-private and public settings follow basic health literacy (HL) standards and guidelines and provide all information to individuals in plain language at the 5th-grade level (Geboers et al., 2018)
	School programs educate students on how to discern misinformation online (Zhao et al., 2023)
	School programs are geared toward students and their families/guardians
	Educational programs that target youth health behavior and HL at the appropriate developmental stage

**Table:**
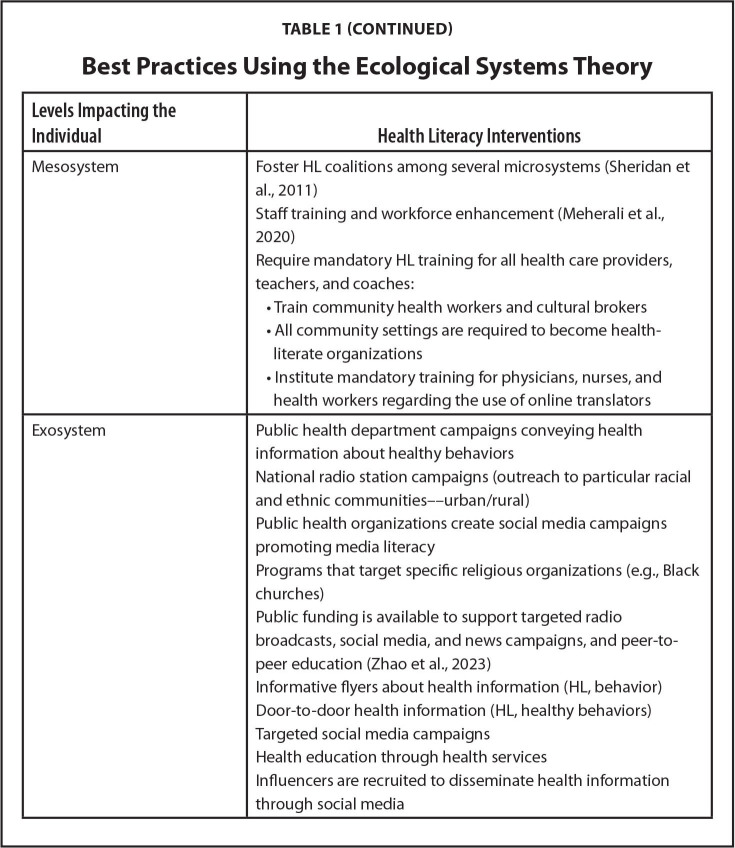


**Levels Impacting the Individual**	**Health Literacy Interventions**

Mesosystem	Foster HL coalitions among several microsystems (Sheridan et al., 2011)
	Staff training and workforce enhancement (Meherali et al., 2020)
	Require mandatory HL training for all health care providers, teachers, and coaches: Train community health workers and cultural brokers All community settings are required to become health- literate organizations Institute mandatory training for physicians, nurses, and health workers regarding the use of online translators

Exosystem	Public health department campaigns conveying health information about healthy behaviors
	National radio station campaigns (outreach to particular racial and ethnic communities––urban/rural)
	Public health organizations create social media campaigns promoting media literacy
	Programs that target specific religious organizations (e.g., Black churches)
	Public funding is available to support targeted radio broadcasts, social media, and news campaigns, and peer-to-peer education (Zhao et al., 2023)
	Informative flyers about health information (HL, behavior)
	Door-to-door health information (HL, healthy behaviors)
	Targeted social media campaigns
	Health education through health services
	Influencers are recruited to disseminate health information through social media

**Table:**
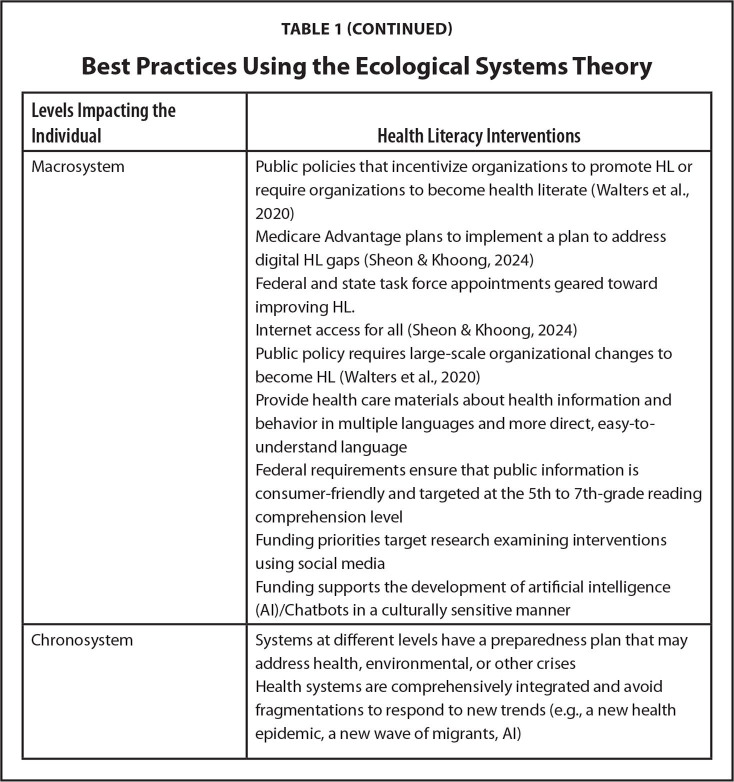


**Levels Impacting the Individual**	**Health Literacy Interventions**

Macrosystem	Public policies that incentivize organizations to promote HL or require organizations to become health literate (Walters et al., 2020)
	Medicare Advantage plans to implement a plan to address digital HL gaps (Sheon & Khoong, 2024)
	Federal and state task force appointments geared toward improving HL.
	Internet access for all (Sheon & Khoong, 2024)
	Public policy requires large-scale organizational changes to become HL (Walters et al., 2020)
	Provide health care materials about health information and behavior in multiple languages and more direct, easy-to-understand language
	Federal requirements ensure that public information is consumer-friendly and targeted at the 5th to 7th-grade reading comprehension level
	Funding priorities target research examining interventions using social media
	Funding supports the development of artificial intelligence (AI)/Chatbots in a culturally sensitive manner

Chronosystem	Systems at different levels have a preparedness plan that may address health, environmental, or other crisesHealth systems are comprehensively integrated and avoid fragmentations to respond to new trends (e.g., a new health epidemic, a new wave of migrants, AI)

## Lessons Learned: Implications

The COVID-19 pandemic highlighted the role of HL in the ability of individuals and communities to understand and follow health guidelines. While there are reports of effective HL interventions among patients with chronic health diseases, the same understanding of successful HL interventions during public health emergencies is poorly understood (Larsen et al., 2022; Mullen et al., 2018). EST is a framework that considers the multiple levels of influence on an individual, including the microsystem, mesosystem, exo-system, macrosystem, and chronosystem. In addition, a core principle underlying EST is that HL is influenced by the complex interplay between the cultural, social, political, and economic environment surrounding the individual.

The insights we learned through the COVID-19 pandemic can inform how we design and implement effective HL interventions during public health emergencies and “normal” times. While the pandemic may be over, AI and the migrant crisis represent the continually changing health environment that must be addressed. For example, AI chatbots offer the potential to provide accessible information in the patient's language; however, the possibility of misuse needs to be assessed (Aggarwal, 2023). The EST is a valuable framework that helps researchers, health care providers, and policymakers meet new challenges and opportunities by emphasizing the importance of a holistic and multidimensional approach.

## Conclusion

The EST highlights the complex relationship between individuals and the different levels of their environments that shape health behavior. Although this paper does not examine the significance of the interventions and best practices, it is imperative for various stakeholders to conduct further research to examine effectiveness and impact. By recognizing the importance of community involvement and interdisciplinary collaborations, health care providers and policymakers can take significant steps to reduce health disparities and promote health equity. It will be increasingly important to structure future HL research to assess the specific individual, familial, societal, and cultural factors to decrease the potential negative impacts of a public health emergency considering the HL environment is constantly evolving. Ultimately, EST serves as a valuable framework for improving HL and interventions, providing a solid foundation for future research and practice in public health.
